# Soil enzyme activities, soil physical properties, photosynthetic physical characteristics and water use of winter wheat after long-term straw mulch and organic fertilizer application

**DOI:** 10.3389/fpls.2023.1186376

**Published:** 2023-05-30

**Authors:** Yonghui Yang, Hao Liu, Jicheng Wu, Sensen Zhang, Cuimin Gao, Shuiqing Zhang, Darrell W. S. Tang

**Affiliations:** ^1^ Institute of Plant Nutrition & Resource Environment, Henan Academy of Agricultural Sciences, Zhengzhou, China; ^2^ International Joint Research Laboratory for Global Change Ecology, School of Life Sciences, Henan University, Kaifeng, Henan, China; ^3^ Yuanyang Experimental Station of Crop Water Use, Ministry of Agriculture, Yuanyang, China; ^4^ Field Scientific Observation and Research Station of Water Saving Agriculture in the Yellow River Basin of Henan Province, Yuanyang, China; ^5^ Henan Provincial Institute of Geology, Zhengzhou, China; ^6^ Soil Physics and Land Management Group, Wageningen University & Research, Wageningen, Netherlands

**Keywords:** straw, soil microbial biomass nitrogen and carbon, organic fertilizer, soil enzymes, photosynthetic physical characteristics, water use efficiency

## Abstract

**Introduction:**

Inappropriate residue and nutrient management leads to soil degradation and the decline of soil quality and water storage capacity.

**Methods:**

An ongoing field experiment has been conducted since 2011 to investigate the effects of straw mulching (SM), and straw mulching combined with organic fertilizer (SM+O), on winter wheat yield, including a control treatment (CK, no straw). We studied the effects of these treatments on soil microbial biomass nitrogen and carbon, soil enzyme activity in 2019, photosynthetic parameters, evapotranspiration (ET), water use efficiency (WUE), and yields over five consecutive years (2015-2019). We also analyzed the soil organic carbon, soil structure, field capacity, and saturated hydraulic conductivity in 2015 and 2019.

**Results:**

Results indicate that compared with CK, SM and SM+O treatments increased the proportion of >0.25mm aggregates, soil organic carbon, field capacity, and saturated hydraulic conductivity, but decreased the soil bulk density. In addition, the SM and SM+O treatments also increased soil microbial biomass nitrogen and carbon, the activity of soil enzymes, and decreased the carbon-nitrogen ratio of microbial biomass. Therefore, SM and SM+O treatments both increased the leaf water use efficiency (LWUE) and photosynthetic rate (Pn), and improved the yields and water use efficiency (WUE) of winter wheat. The combination SM (4.5 t/ha)+O (0.75 t/ha) was more effective than SM alone, and both treatments were superior to the control.

**Conclusion:**

Based on the results of this study, SM+O is recommended as the most effective cultivation practice.

## 1 Introduction

Water scarcity and farmland degradation due to inappropriate residue and nutrient management are major influencing factors on agricultural production globally, especially in eastern Henan province, China. This has led to high evaporation rates and low soil moisture retention capacity, which has aggravated the imbalance between water supply and crop water demand (Hemmat and Eskandari, 2004; Vita et al., 2007), leading to declines in wheat production. Therefore, ameliorating soil quality, improving soil structure, increasing soil water storage and soil moisture conservation capacity, and improving water use efficiency and crop yields have become important issues concerning agricultural practices.

Suitable field practices not only ensure stable and high agricultural yields (Giller et al., 2009) but also benefit the sustainability of ecological environment (Knowler and Bradshaw, 2007). Therefore, it is important to discuss the impacts of field practices on the soil, crop yields and crop growth conditions. Crop straw is an important agricultural resource, which can also provide organic acids and neutralize the alkalinity of the soil and increase soil organic carbon, which then improves soil quality. Straw mulching also increases water use efficiency and the sustainability of agriculture (Hobbs et al., 2008), and has therefore been widely used in crop cultivation (Kurothe et al., 2014; Zhao et al., 2016; Alliaume et al., 2017).

In addition, straw addition can increase soil permeability and soil fertility, preserve soil moisture, and inhibit weed growth. Straw mulching or straw returning can also regulate soil temperature and moisture (Gan et al., 2013), decrease erosion (Bhatt and Khera, 2006), increase soil organic carbon (Dabney et al., 2001; Liu et al., 2014) and soil infiltration, and improve the soil’s structure while decreasing its bulk density (Choudhury et al., 2014). Lenka and Lal (2013) found that a lack of straw incorporation in soils will eventually result in low soil organic carbon levels. Lugato et al. (2006) found that straw incorporation led to substantially greater soil organic carbon stocks than straw removal under various nitrogen application rates for 40 years. Combining straw application with other farming techniques such as organic fertilizer that also improves the soil may lead to overall better soil quality, and thereby enhance crop yields and promote soil sustainability.

The use of excessive chemical fertilizer leads to soil compaction and soil acidification, which in turn reduces soil quality and productivity. However, application of organic manure or fertilizer may improve the quality of the soil and the characteristics of soil aggregates, and enhance soil moisture, which improves crop growth, yield and water use efficiency (Karami et al., 2012; Liu et al., 2013; Wang et al., 2019). In addition, long-term application of organic fertilizer improves photosynthetic capacity, extends leaf aging (Dordas, 2009; Bogard et al., 2010), improves soil enzyme activities (Rehana et al., 2008; Pu et al., 2020) and increases soil microbial carbon and nitrogen (Elfstrand et al., 2007; Liang et al., 2010; Nakhro and Dkhar, 2010). However, Zhang et al. (2013) found that a long-term application of organic fertilizer alone had no obvious effect on soil microbial carbon content, but positive effects were observed when combined with inorganic fertilizer incorporation. Yang et al. (2022) found that applying organic fertilizer combined with subsoiling tillage can improve soil structure and soil organic carbon, increase soil microbial nitrogen and carbon, improve the activity of soil enzymes, and thus boost yields and water use efficiency (WUE). Yang et al. also (2021) found that straw mulching coupled with subsoiling can improve the photosynthetic characteristics and increase the yield and WUE of crops. In addition, Yang et al. (2018) found that short-term application of straw mulch coupled with organic fertilizer could increase soil porosity and field moisture capacity. However, the effects of long-term application of straw mulching combined with organic fertilizer on soil properties, soil microbial nitrogen and carbon, soil enzyme activity, photosynthetic characteristics, evapotranspiration (ET), and wheat yields still requires further study.

We hypothesize that straw mulching coupled with organic fertilizer leads to better effects on soil properties, soil microbial nitrogen and carbon content, the activity of soil enzymes, photosynthetic physical characteristics, yields, and the ET of wheat, than straw mulching alone. This may help in reducing or resolving crop losses due to the decline of soil water storage and moisture holding ability, caused by tillage and soil degradation induced by poor fertilization practices. Therefore, based on 5 years (from 2015 to 2019) of observations from an experiment that has been in progress since 2011, we study the influence of straw mulching only, and that of straw mulching and organic fertilizer in combination, on soil properties and the WUE and yield of wheat, while also investigating the mechanisms of efficient crop ET and crop yield increases related to these practices. This will facilitate a better fundamental understanding of the effects and mechanisms-of-action of such agricultural practices, and its potential for wider adoption in geographically similar regions.

## 2 Materials and methods

### 2.1 Experimental location

The experimental site is located at Tongxu (144°26*’*58.47*’’*E; 34°25*’*44. 26*’’* N, 62m AMSL, average annual precipitation 675.9 mm, average annual temperature 14.2 °C). Approximately 60% of rainfall occurs between July and September. The terrain is flat and the soil fertility is uniform. The experiment has been conducted since October 2011, and five years (2015 to 2019) of relevant data were collected for this study. Under the ISSS system for classifying soil textures (Rousseva, 1997), the soil in the field is heavily sandy and loamy. Other pertinent physical and chemical properties of the soil are shown in **Table 1**. Wheat-maize rotation has been applied to this area for over five decades.

**Table 1:** The soil total nitrogen, nitrate nitrogen, ammonium nitrogen, available phosphorus, available potassium, bulk density, and soil organic matter in top (0-20 cm) layer of soil.

Factors	Soil total nitrogen/g·kg^-1^	Nitrate nitrogen/mg·kg^-1^	Ammonium nitrogen/mg·kg^-1^	Available phosphorus/mg·kg^-1^	Available potassium/mg·kg^-1^	Bulk density/g·cm^-1^	Soil organic matter/g·kg^-1^
Value	0.81	74.31	55.89	19.82	90.31	1.31	11.42

### 2.2 Experimental design

In this experiment, the treatments are CK (control, conventional tillage to a depth of 15 cm using a rotavator plow), SM (4.5 t/ha straw mulching covered two weeks after wheat emergence, conventional tillage), and SM+O (4.5 t/ha straw mulching combined with 0.75 t/ha organic fertilizer, straw mulching covered two weeks after wheat emergence, conventional tillage). The experiment was designed with randomized split-plots, and the field was divided into 9 plots. Thus, each treatment had three replicates. Each plot had an area of 33.6 m ^2^ (5.6 m × 6 m), with wheat spaced 20 cm apart, sown at 0.195 t/ha. The organic fertilizer had NPK contents of 1.5%, 1.2%, and 0.8%, respectively, and identical total amounts of N (0.225 t/ha), P (0.105 t/ha), and K (0.075 t/ha) were applied under each treatments. Half the N-fertilizer was applied before wheat sowing. The remainder was applied at the jointing (30%) and booting (20%) stages. 60mm of total irrigation was done during the jointing and grouting stages. The wheat cultivar used was Aikang 58. Wheat was sown between 20th and 25th October and harvested around June 1st.

**Figure 1:**
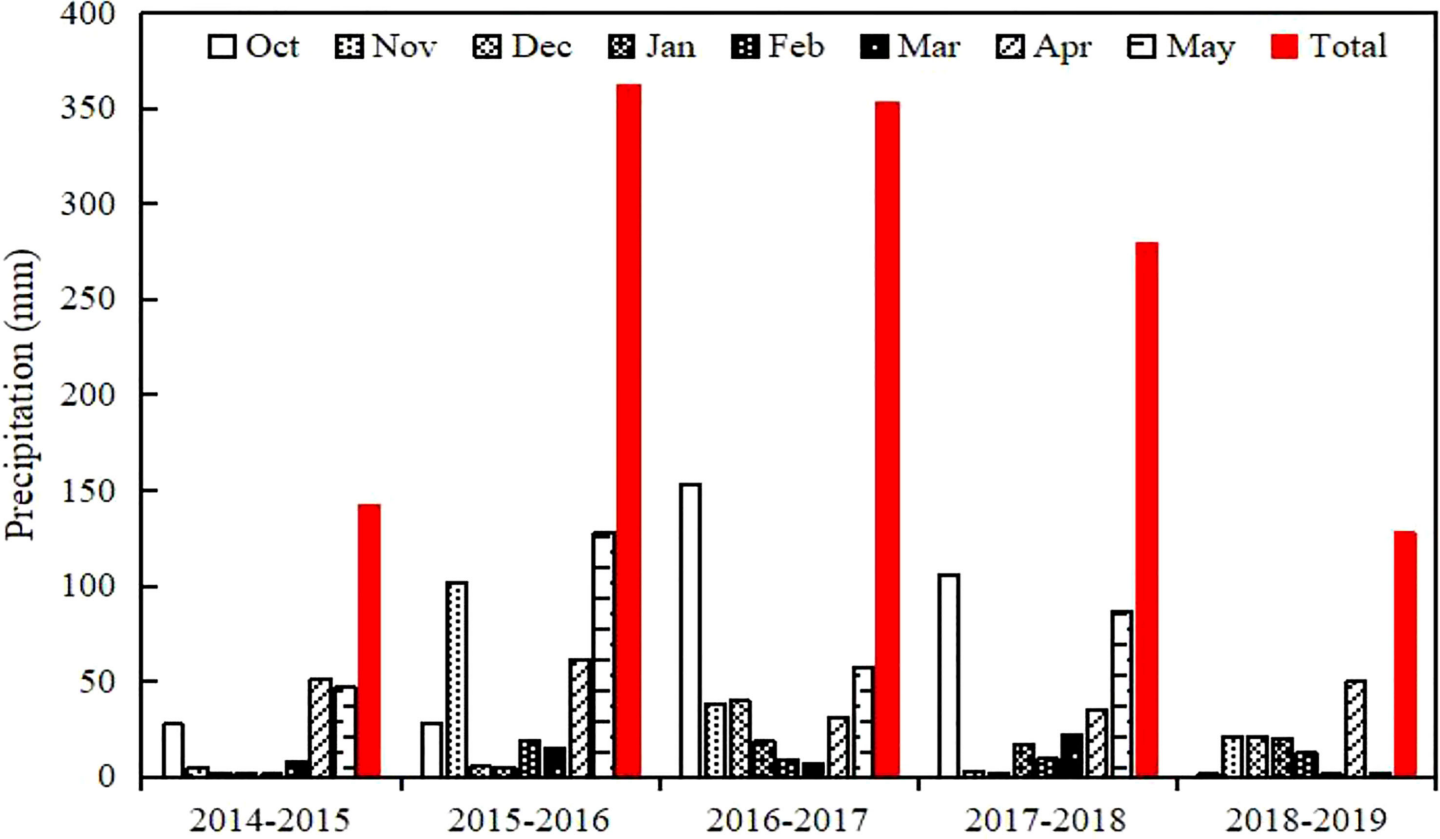
Precipitation during the growing period of winter wheat in five seasons.

The precipitation rates in **Figure 1** occurred between 2014 and 2019 during the wheat season, and indicates the rainy seasons of 2015-2016 and 2016-2017, and the dry seasons of 2014-2015 and 2018-2019.

### 2.3 Sampling and measurements

#### 2.3.1 Photosynthetic physiological parameters

On sunny and windless mornings (9:30–11:00 AM) during the filling stage of wheat, the net photosynthetic rate (Pn), transpiration rate (Tr) and stomatal conductance (Gs) under each treatment were measured with 9 data replicates. The leaf water use efficiency (LWUE) was measured with the following equation (additional detail in Fischer and Turner, 1978 and Powel, 1984):


(1) LWUE=Pn/Tr

(LWUE, µmol CO_2_ mmol^−1^ H_2_O; Tr, mmol H_2_O (m^2^·s)^−1^; Pn, µmol CO_2_ (m^2^·s)^−1^)

#### 2.3.2 Evapotranspiration

The applicable soil water balance in this study is (Wang et al., 2019; Lenka et al., 2020).


(2) ET=(P+I+G+ΔW)−(R+D)

where ET (mm) is the evapotranspiration, and *I* and *P* represent the irrigation and precipitation, respectively (mm), *ΔW* is the change in soil water storage in the soil at 0-100 cm depth between sowing and harvest stage of winter wheat (mm). *D*, *R* and *G* represent drainage, surface runoff and capillary rise from groundwater. No significant drainage from the upper 100 cm of soil, nor surface runoff, were observed in our study area. As the depth of the water table was greater than 10 m, groundwater does not contribute to root zone moisture.

#### 2.3.3 Crop yields and water use efficiency

At the harvest stage, a 4m^2^ subregion of each plot was randomly chosen. Crops from these subregions were then harvested, air-dried, and weighed, in order to calculate the area-normalized grain yield. The water use efficiency (WUE) of the crop is defined as:


(3) WUE=Y/ET

where Y is the grain yield.

#### 2.3.4 Soil physico-chemical properties and biological activity

Extraction by fumigation with chloroform (Vance et al., 1987; Christianson, 1988; Moore et al., 2000) was employed to quantify the soil microbial biomass nitrogen and carbon. Activities of cellulase, sucrase, protease and urease in the soil were determined by the method of Qin et al. (2020). The total content of soil organic carbon was also determined, following Walkley and Black (1934) and Westerman (1990). The size distribution of water-stable aggregates (> 0.25 mm) was obtained with the wet-sieve method (Elliot, 1986). Available phosphorus was determined with the method of De Silva et al. (2015). Available potassium, nitrate nitrogen and ammonium nitrogen were determined with the method of Ove (1989).

### 2.4 Statistical analysis

**Figure 2:**
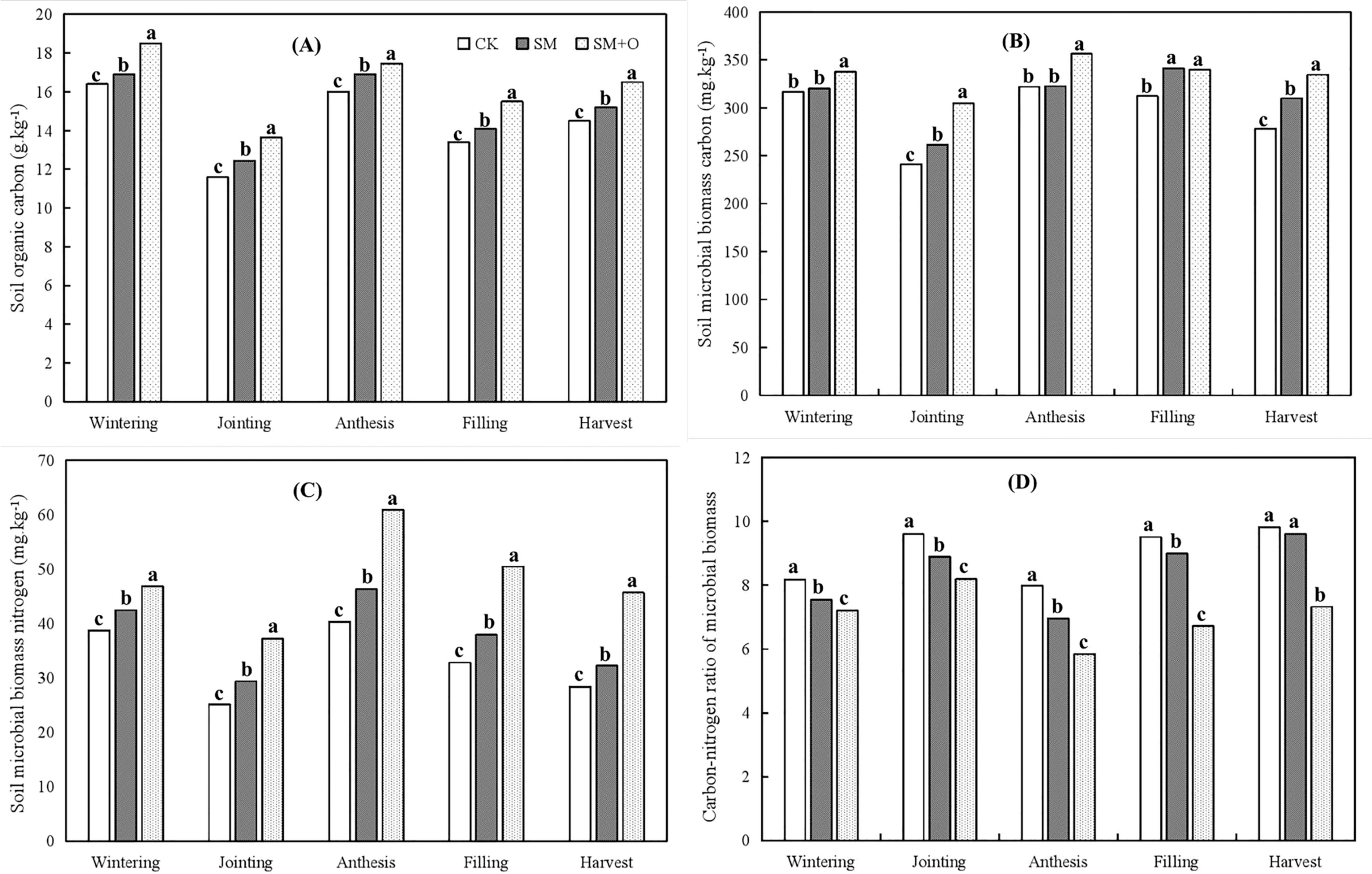
Effects of SM and SM+O treatments on soil organic carbon **(A)**, soil microbial biomass carbon **(B)** and nitrogen **(C)** and carbon-nitrogen ratio of microbial biomass **(D)** at different growth stages of winter wheat in 2019. CK, conventional tillage; SM, straw mulching; SM+O, straw mulching with organic fertilizer. Different lowercase letters in the same growth stage indicate significant differences among treatments by LSD test (*P*< 0.05).

**Figure 3:**
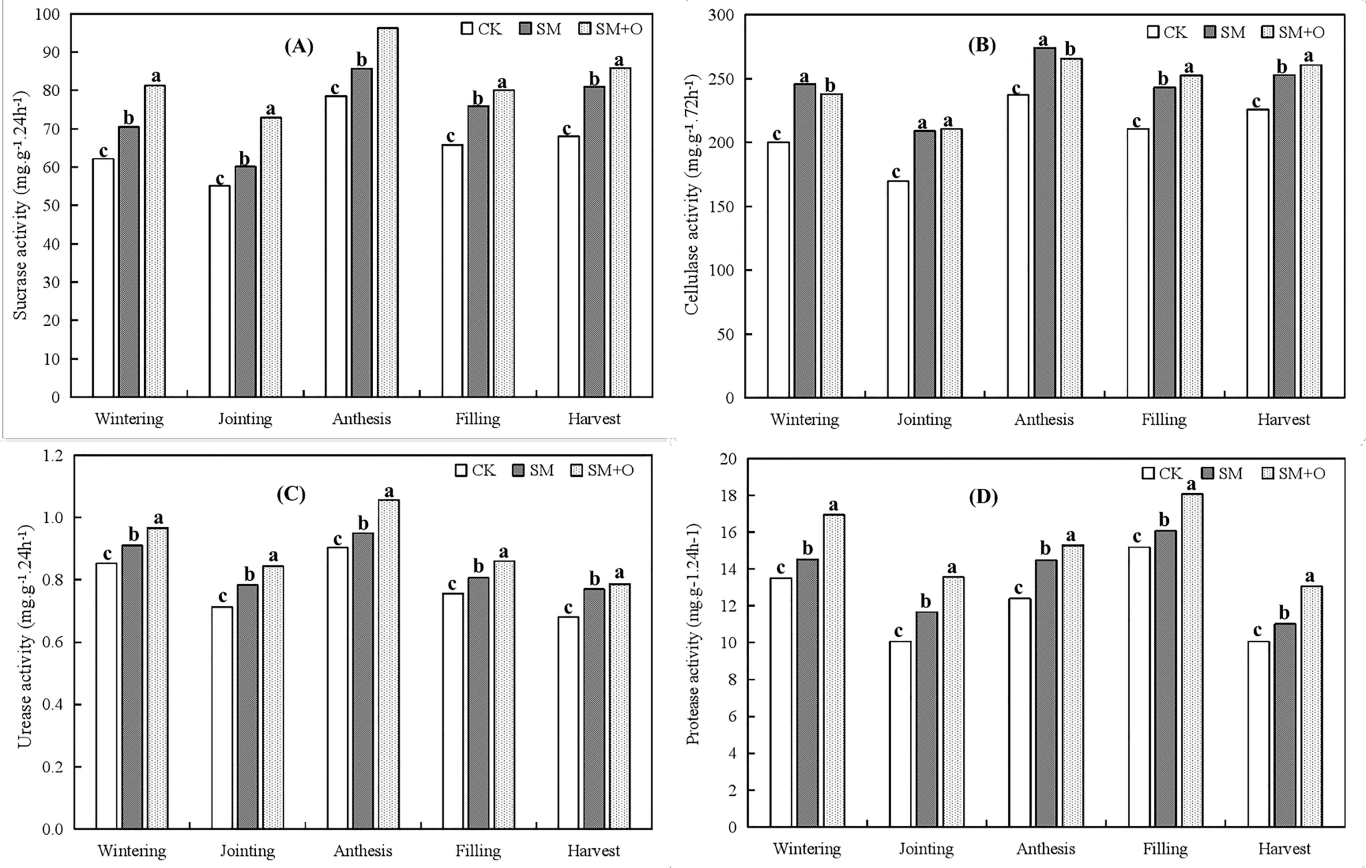
Effects of SM and SM+O treatments on sucrase activity **(A)** and cellulase activity **(B)**, urease activity **(C)** and protease activity **(D)** of soil at different growth stages of winter wheat in 2019. CK, conventional tillage; SM, straw mulching; SM+O, straw mulching with organic fertilizer. Different lowercase letters in the same growth stage indicate significant differences among treatments by LSD test (*P*<0.05).

**Figure 4:**
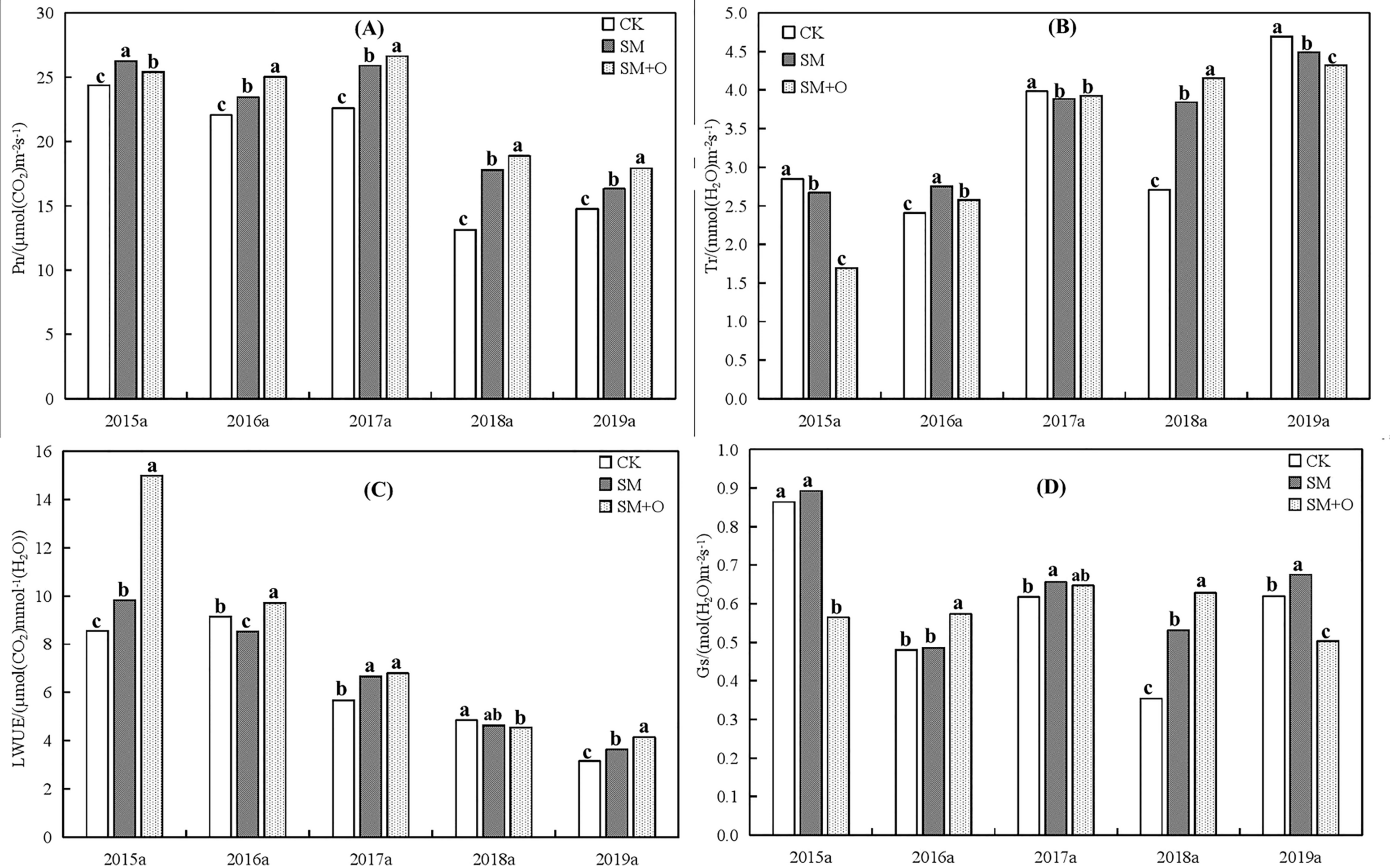
Effects of SM and SM+O treatments on Pn **(A)**, Tr **(B)**, LWUE **(C)**, Gs **(D)** at filling stages of winter wheat in different years. CK, conventional tillage; SM, straw mulching; SM+O, straw mulching with organic fertilizer; Pn, net photosynthetic rate; Tr, Transpiration rate; LWUE, leaf water use efficiency; Gs, stomatal conductance. Different lowercase letters in the same year indicate significant differences among treatments by LSD test (*P*<0.05).

**Figure 5:**
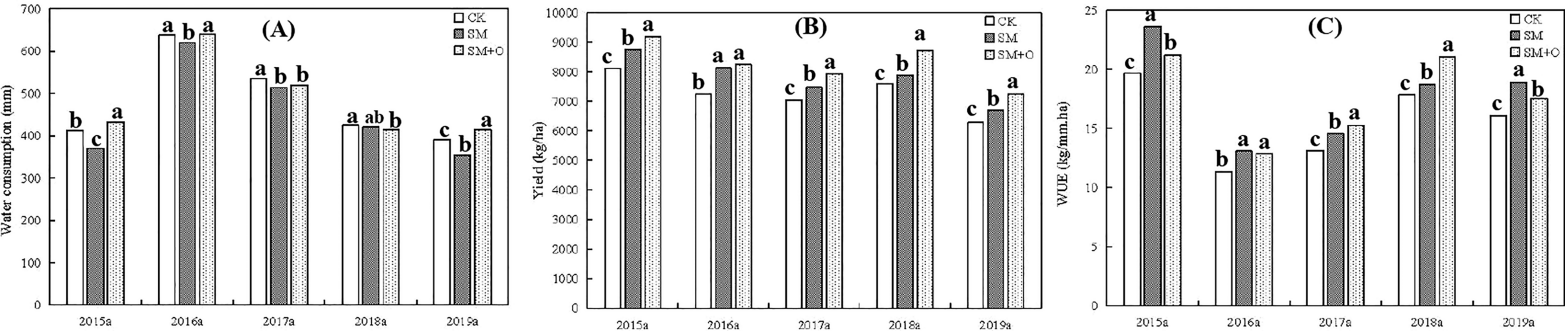
Effects of SM and SM+O treatments on yield **(A)**, water consumption **(B)** and water use efficiency (WUE) **(C)** at different growth stages of winter wheat in the three seasons of 2015-2019. CK, conventional tillage; SM, straw mulching; SM+O, straw mulching with organic fertilizer. Different lowercase letters in the same year indicate significant differences among treatments by LSD test (*P*<0.05).

Statistical analyses of data with three replicates were carried out with SPSS 21.0. The effects of the various treatments were compared with ANOVA, at significance levels of *P*<0.05 (**Table 2**; **Figures 2**–**5**). The relationships between the various observed experimental outcomes were analyzed with linear regression, and their coefficients are listed in **Table 3**.

**Table 2:** Field water capacity, bulk density, >0.25mm aggregate and saturated hydraulic conductivity under different treatments in different years.

Treatments	Field water capacity/(%)		Bulk density /(g/cm3)		>0.25mm aggregate /(%)		Saturated hydraulic conductivity/(mm/h)	
	2015	2019	2015	2019	2015	2019	2015	2019
CK	17.72Bc	18.31Ac	1.41Aa	1.38Aa	55.64Ac	56.77Ac	0.99Ac	1.03Ab
SM	18.10Bb	19.83Ab	1.36Aab	1.30ab	60.12Bb	64.71Ab	1.62Bb	2.15Aa
SM+O	19.35Ba	21.97Aa	1.30Ab	1.21Bb	64.23Ba	71.79Aa	1.95Ba	2.95Aa

CK, conventional tillage; SM, straw mulching; SM+O, straw mulching with organic fertilizer. Different uppercase letters in the line in same parameter indicate significant differences among treatments by LSD test (*P*< 0.05). Different lowercase letters in the column indicate significant differences among treatments by LSD test (*P*< 0.05).

**Table 3:** Relationships among water consumption, WUE and yield of wheat, soil organic carbon, soil microbial biomass carbon and nitrogen, and soil enzyme activity, photosynthetic parameters and soil physical properties.

Factors	Carbon-nitrogen ratio of microbial biomass	Soil organic carbon	Soil microbial biomass carbon	Soil microbial biomass nitrogen	Sucrase activity	Cellulase activity	Urease activity	Protease activity	Pn	Tr	LWUE	Gs	Field water capacity	Bulk density	>0.25mm aggregate	Saturated hydraulic conductivity
Yield	-0.943*	0.998**	0.985**	0.980**	0.937*	0.917*	0.898*	0.995**	0.995**	-0.986*	0.995**	-0.729*	1.000**	-0.995*	0.991**	0.910*
ET	-0.740*	0.531	0.311	0.640	0.135	0.082	0.036	0.560	0.382	-0.319	0.384	-0.948*	0.467	-0.379	0.348	0.064
WUE	-0.079	0.345	0.561	0.216	0.701*	0.738*	0.768*	0.312	0.497	-0.554	0.495	0.327	0.413	-0.500	0.528	0.750*

*P< 0.05; **P< 0.01. ET, evapotranspiration; WUE, water use efficiency; Pn, net photosynthetic rate; Tr, transpiration rate; LWUE, leaf water use efficiency; Gs, stomatal conductance.

## 3 Results

### 3.1 Soil physical properties

In **Table 2**, it is shown that the field water capacity, proportion of water-stable macro aggregate (>0.25mm) and saturated hydraulic conductivity in 2019 were all significantly (*P<*0.01) higher than those in 2015 and that the soil bulk density in 2019 was lower than that in 2015. Compared with CK, SM and especially SM+O treatment increased the field capacity, proportion of water-stable macro aggregates, and saturated hydraulic conductivity, but reduced the soil bulk density.

### 3.2 Soil organic carbon, microbial biomass carbon and nitrogen

**Figure 2** shows that SM and SM+O treatments increased soil organic carbon, soil microbial biomass carbon, and soil microbial biomass nitrogen, but decreased the carbon-nitrogen ratio of microbial biomass. Once again, SM+O treatment had the largest effect, followed by SM.

### 3.3 Soil enzyme activity

**Figure 3** shows that across all growth stages, SM and SM+O treatments increased soil sucrase activity, cellulase activity, protease activity and urease activity. SM+O treatment had the best effect on soil protease, sucrase, and urease activity, followed by SM and CK. Cellulase activity under SM+O treatment was the highest at all growth stages, except at the wintering and anthesis stages of wheat, where cellulase activity under SM treatment was the highest.

### 3.4 Photosynthetic physiological characteristics

**Figures 4A**, **B** show that the net photosynthetic rate (Pn) of wheat decreased from 2015 to 2019, whereas the transpiration rate (Tr) gradually increased. Pn under SM+O treatment after 2016 was highest, followed by SM and then CK. In 2015, Pn in decreasing order occurred under SM > SM+O > CK treatments. Tr under CK in 2015, 2017 and 2019 was the highest, and Tr under SM+O treatment was the lowest. However, in 2018, the results were the opposite, and Tr under SM+O treatment was the highest. In 2016, the Tr after SM treatment was significantly higher than in the other treatments, followed by SM+O and then CK. Due to the differences between Pn and Tr under different treatments, the leaf water use efficiency (LWUE) also differed across treatments. **Figure 4C** shows that, from 2015 to 2019, the leaf water utilization efficiency showed a gradually decreasing trend. LWUE after SM+O treatent was highest across all years except for 2018. In 2018, the LWUE under SM+O treatment was the lowest compared with SM and CK treatments. As shown in **Figure 4D**, in 2016 and 2018, SM+O treatment significantly (*P<*0.01) increased stomatal conductivity (Gs) of wheat leaves as compared to SM and CK treatments. However, in 2015 and 2019, the Gs of wheat leaves under SM+O treatment was the lowest.

### 3.5 ET, yield and water use efficiency

**Figure 5A** shows that ET of wheat increased initially (2016) and then decreased gradually from 2017 to 2019. In 2016, ET of wheat was higher than in other years (*P<*0.01), and SM treatment reduced the ET of wheat compared with CK. In 2015 and 2019, the ET of wheat under SM+O treatment was higher than CK (*P<*0.01), but that under SM treatment was still lower than CK (*P<*0.01). In 2016 and 2017, the ET of wheat under SM and SM+O treatments were lower than CK (*P<*0.01). From **Figure 5B**, the yield of wheat decreased gradually from 2015 to 2019. SM and SM+O treatments increased the yield of winter wheat in the various years, and yields were ranked as SM > SM+O > CK (*P<*0.01). The ET of wheat varied over the years. **Figure 5C** shows that SM and SM+O treatments significantly (*P<*0.01) increased the water use efficiency of wheat (WUE). WUE in 2015 was the highest compared with other years, at more than 20 kg/(mm^·^ha), and WUE under SM treatment in 2016 and 2019 was the highest, followed by SM+O and CK. The WUE in 2016 and 2017 were lower than in other years at 11.3 to 15.2 kg/(mm^·^ha). In 2017 and 2018, the WUE under SM+O treatment was the highest, followed by SM and CK.

### 3.6 Correlation analysis

The relationships between the ET of wheat, WUE, and yield, along with the soil properties including organic carbon content, soil microbial biomass nitrogen and carbon, the activities of soil enzymes, soil physical parameters and photosynthetic parameters, are shown in **Table 3**. Significant (P<0.05) or very significant (P<0.01) positive correlations were found between wheat yield and the following: soil microbial biomass nitrogen and carbon, soil organic carbon, the activities of sucrase, cellulase, urease, and protease, LWUE, Pn, field capacity, the fraction of aggregates >0.25 mm, and the saturated hydraulic conductivity. Significant (P<0.05) negative correlations were found between wheat yield and the carbon-nitrogen ratio of microbial biomass, Tr, Gs, and bulk density. Significant (P<0.05) negative correlations were found between ET and the carbon-nitrogen ratio of microbial biomass and Gs. WUE was significantly (P<0.05) positively correlated with the activities of sucrase, cellulase, and urease, and the saturated hydraulic conductivity of the soil.

## 4 Discussion

### 4.1 Soil properties, microbial biomass nitrogen and carbon, and the activities of soil enzymes

Reductions in soil organic carbon and soil quality due to inappropriate or excessive tillage or inappropriate fertilizer use could lead to exacerbated soil degradation and reduced soil porosity, water conductivity and effective water content, the growth of crops (Spiegel et al., 2007). Application of straw mulching and organic fertilizer are traditional, widely used practices that, alone or combined, can increase soil organic carbon, protect the soil and improve its structure. Zhao et al. (2018) found that straw returning significantly increased the soil organic carbon in aggregates of various particle sizes, and the proportion of macroaggregates at 0-20 cm depth. Straw mulching combined with organic fertilizer was found to improve the structure and increase the field moisture capacity in cinnamon soil (Yang et al., 2018). In fluvo-aquic soil, we found that compared with CK, SM and especially SM+O treatments more greatly increased the proportion of macroaggregates (> 0.25 mm), which was conducive for increasing soil organic carbon (Mikha and Rice, 2004) and improving stable aggregates (Song et al., 2019) in more stable soil structures (Six et al., 2000).

Furthermore, straw can directly prevent raindrops from impacting the soil surface after tillage, thus reducing soil erosion, protecting macropore structures, decreasing soil bulk density, increasing soil infiltration, and allowing more water to percolate to deeper soil. This promotes crop root growth, especially when combined with organic fertilizer, which then improves soil structure, field capacity and saturated hydraulic conductivity, facilitating the retention and transport of water. The above indicates that SM+O is a good practice for improving soil structure and soil water retention, and provides a better soil environment for crop growth compared to CK.

Soil material conversion and the energy cycle are closely related to soil organic carbon content, and microbial biomass nitrogen and carbon (Nsabimana et al., 2004). Li et al. (2017) found that organic fertilizer applied a single time can enhance microbial biomass carbon during crop growth. Pi et al. (2017) found that short-term application of straw mulching decreases soil organic carbon content, and microbial biomass nitrogen and carbon, while releasing little organic carbon. In the present study, changes in soil microbial biomass carbon and nitrogen content were found to be consistent with trends in soil organic carbon changes. SM+O treatment resulted in the highest levels of soil microbial biomass nitrogen and carbon. This shows that in comparison with straw application or conventional tillage, combining straw mulching with organic fertilizer improved soil physical and chemical properties, promoted root growth and nutrient absorption, and increased the generation of root secretions, which enhanced soil microbial biomass quantity and soil microbial activity (Kuzyakov and Xu, 2013; Yu, 2015). In addition, straw mulching combined with organic fertilizer was more conducive to reducing the amount of carbon to nitrogen ratio of microbial biomass, which may increase microbial activity and promote straw decomposition, thus, releasing nutrients for crop growth. At the same time, improved crop drought tolerance and enhanced the utilization rate of water and fertilizer.

Soil enzymes are important indicators for measuring soil nutrient levels and nutrient circulation (Sinsabaugh et al., 2008). Straw mulching could significantly improve the activities of urease, sucrase, protease and alkaline phosphatase (Fontaine et al., 2003.) in the soil, while organic fertilizer could improve the activities of urease, sucrase, and cellulase in the soil (He et al., 2020). This study shows that SM+O raised the activities of urease, cellulase and sucrase in the soil to a greater extent than straw mulching alone. These indicate that straw mulching mechanically makes the soil more porous (Yang et al., 2018) and improves the distribution of soil organic matter while facilitating enzyme activity (Balota et al., 2004). At the same time, organic fertilizers boost soil organic carbon, root exudate content and microbial activity, which increases the activities of soil enzymes (Rehana et al., 2008; Pu et al., 2020). Hence, straw mulching coupled with organic fertilizer (SM+O) improved the activities of urease, cellulase and sucrase in the soil, which benefitted crop growth. The above indicates that optimal soil physical and chemical properties could improve the activity of soil enzymes (Wei et al., 2013). In this regard, SM+O treatment was more effective than SM and CK treatments.

### 4.2 Photosynthetic physiological characteristics

Improved soil structure promotes soil moisture availability and physiological function (Xiao et al., 2007; Lamptey et al., 2020). Straw mulching is an effective measure to improve soil properties, increase soil water, boost leaf area and enhance the net photosynthetic rate (Pn) of wheat (Yang et al., 2014; Zhang et al., 2019), which then improves crop growth and yields. In our study, we found that the Pn of SM treatment in 2015 was higher than that of other treatments (*P<*0.01). However, Pn under SM+O treatment was highest amongst treatments in 2016, 2017, 2018 and 2019. These indicate that straw mulching allowed soil to retain more rainfall, which inhibited soil water loss and improved Pn. Furthermore, the combined effect of straw cover and organic fertilizer was more conducive to soil water retention and photosynthesis. Pn in 2015, 2016 and 2017 were higher than that in 2018 and 2019, especially in 2015, when rainfall was significantly less than in other years (*P<*0.01). These show that less rainfall leads to low soil moisture, which improved the photosynthetic rate of crops (Zhang et al., 2008), though these results may depend on climate and crop varieties (Lamptey et al., 2020).

However, the trend of Pn variation was not consistent with that of Tr. In 2015, 2017 and 2019, SM and especially SM+O treatments decreased the Tr of wheat, and thereby increased leaf water use efficiency (LWUE). Thus, SM+O treatment resulted in the highest LWUE in those years. Tr under SM and SM+O treatments increased in 2016 and 2018, which should not have had beneficial effects on LWUE. However, in 2016, SM+O treatment still boosted the LWUE of wheat. This is because the effect of the Pn increment was more significant than that of Tr, so the overall effect was an improved LWUE and increased dry matter (Shi et al., 2016).

Most studies showed that the stomatal conductance (Gs) is positively correlated with the photosynthetic and transpiration rates (Yao et al., 2010; Zhang et al., 2020). However, it was not that the greater the Gs, the better, as there is an optimum Gs at which the LWUE is maximized. Farquhar and Sharkey (1982) found that low Gs resulted in low net photosynthesis rates (Pn), as that restricts CO_2_ uptake, whereas high Gs led to higher Pn but at the expense of water loss via transpiration (Leakey et al., 2009; Blatt and Lawson, 2014). We found that SM+O treatment significantly improved the Pn and LWUE of wheat in different years, while the Tr of wheat was maintained at a low level compared with the CK and SM treatments, except in 2018. However, in 2018, high Gs leads to high Pn and Tr, whereas LWUE was lower than the CK (*P<*0.01) suggesting that Gs plays a key role in water use. The Gs effect seems to depend on soil moisture, temperature (Lenka et al., 2020) and relative air humidity (Zhang et al., 2020), nevertheless, further research is required to better understand its role.

The above indicates that an optimal stomatal conductivity minimizes the loss of water, and improves the photosynthetic rate, which then increases the accumulation of dry plant mass per unit of water. SM+O treatment is thus an effective practice for improving the photosynthetic physiological characteristics of crops, and promoting an increase in photosynthetic efficiency.

### 4.3 ET, yield and WUE

Straw mulching and organic fertilizer treatment led to increased organic carbon and improvement of soil microbial communities (Bending et al., 2002; Ji et al., 2014; Gu et al., 2019), betterment of the soil and distribution of soil pores (Yang et al., 2013; Yang et al., 2018) and increased soil moisture. Thus, it provided favorable environmental conditions for improved crop yields and WUE (Hou et al., 2012; Yang et al., 2018).

However, straw mulching in some cases could also lead to a negative effect (Li et al., 2008; Balwinder et al., 2011); especially if excessively applied. Appropriate straw mulching levels significantly improved photosynthetic physiological characteristics and the micro-ecological environment of rhizosphere soil, which then increases crop yield. However, straw mulching at high levels had a negative impact on crop growth (Zhang et al., 2015), because excess straw causes microorganisms to consume too much soil nitrogen, resulting in nutrient imbalance and reduced crop yield.

In our study, we found that SM and SM+O treatments increase the yield of wheat compared with CK, because straw contains nutrients that could be used by the crops (Li et al., 2004). SM+O treatment resulted in the highest yield of wheat because when organic fertilizer was combined with straw, it supplemented nitrogen for the process of straw decomposition, and prevented microbes from competing with crops for soil nutrients when decomposing straw. Therefore, the addition of organic fertilizer under straw mulching not only promotes straw decomposition, but ensures nutrient balance, which facilitates crop growth.

In addition, SM and SM+O treatments also increased the WUE of wheat. Compared with CK and SM, SM+O treatment led to lower ET and increased the WUE of wheat in 2017 and 2018. In addition, in 2015, 2016 and 2019, low ET of wheat under SM+O treatment also resulted in the highest WUE of wheat in these three years. This indicates that optimal straw mulching improved the growing environment for the crops, which increased yields and WUE (Ocio et al., 1991; Gao et al., 2019; Zhang et al., 2019), especially when combined with organic fertilizer.

Rainfall had a large impact on crop water use. The years with less rainfall (2015 and 2019) led to larger increases in the WUE of wheat grain compared to the other years (2016, 2017 and 2018), especially under the combination of straw mulching and organic fertilizer, which indicates that straw mulching and organic fertilizer were effective at reducing yield losses in a dry climate with moisture limited crop growth. This indicates that during dryer years, ongoing long-term regimes of SM and SM+O application were better able to retain more moisture in the soil, and improve photosynthetic capacity and photosynthetic product accumulation (dry matter), while relieving drought stress in crops, which then increased yields and WUE. It follows that the impacts of organic fertilizer coupled with straw mulching, on grain yield and WUE, greatly depends on the rainfall in a particular year (Wang and Shangguan, 2015; Zhang et al., 2017). We have shown that SM+O is a superior alternative to burning crop straw to increase soil organic carbon in the field, as it avoids the environmental issues and climate impacts associated with the latter (Sun et al., 2016).

Organic fertilizers are rich in nitrogen, and are thus useful for improving the carbon-nitrogen ratio of straw and promoting straw decomposition. Therefore, compared with straw mulching alone, straw mulching combined with organic fertilizer causes the release of more nutrients, which can be taken up by crops, thus increasing yields. In addition, the long-term application of straw mulching coupled with organic fertilizer leads to stable soil physical properties (Dossou-Yovo et al., 2016) and regulates soil moisture (Zhang et al., 2015), nutrients, temperature, and microbal community, which results in better crop physiological characteristics, promotes the accumulation of dry matter (Shi et al., 2016), and improves crop yields.

## 5 Conclusions

The long-term application of SM or SM+O treatments increased soil organic carbon content and microbial biomass nitrogen and carbon, stimulated an improvement in cellulase, sucrase and urease activity, and improved the structure of the soil. These increased the net photosynthetic rate and LWUE of wheat, which then increased the yield and WUE of wheat, especially in years with less rainfall. Compared with SM, long-term SM+O treatment led to superior yields and WUE for wheat, as the combination provides soil microbes with sufficient nutrients and prevents them from competing with crops for bioavailable nutrients in the soil. Therefore, appropriate long-term application of SM+O may be optimal for regulating the ET of wheat. Correlation analyses indicate that crop yields are significantly positively correlated with soil organic carbon, soil microbial biomass nitrogen and carbon, soil enzymatic activities (sucrase, protease), Pn, LWUE, soil physical properties (Field capacity, >0.25mm aggregate, saturated hydraulic conductivity), and the activities of soil enzymes (sucrase, cellulase, and urease). The combined application of SM+O benefits crop yields through its positive effects on the above soil physical and ecological properties.

## Data availability statement

The raw data supporting the conclusions of this article will be made available by the authors, without undue reservation.

## Author contributions

YY wrote the main manuscript. HL wrote the part of the manuscript and revised the manuscript. JW revised and gave some advice for the manuscript. SeZ prepared the figure and table of the manuscript. CG and ShZ performed most of the experiments. DT edited the language and modified the main manuscript. All authors contributed to the article and approved the submitted version.
